# Transcriptomic and Ultrastructural Signatures of K^+^-Induced Aggregation in *Phytophthora parasitica* Zoospores

**DOI:** 10.3390/microorganisms8071012

**Published:** 2020-07-07

**Authors:** Ilaria Bassani, Corinne Rancurel, Sophie Pagnotta, François Orange, Nicolas Pons, Kevin Lebrigand, Franck Panabières, Laurent Counillon, Xavier Noblin, Eric Galiana

**Affiliations:** 1 Université Côte d’Azur, INRAE, CNRS, ISA, 06903 Sophia Antipolis, France; corinne.rancurel@inrae.fr (C.R.); franck.panabieres@inrae.fr (F.P.); 2 Université Côte d’Azur, Centre Commun de Microscopie Appliquée, 06108 Nice, France; sophie.pagnotta@unice.fr (S.P.); francois.orange@unice.fr (F.O.); 3 Université Côte d’Azur, CNRS, IPMC, 06560 Sophia Antipolis, France; pons@ipmc.cnrs.fr (N.P.); lebrigand@ipmc.cnrs.fr (K.L.); 4 Université Côte d’Azur, CNRS, LP2M, 06107 Nice, France; laurent.counillon@unice.fr; 5 Université Côte d’Azur, CNRS UMR 7010, Institut de Physique de Nice, 06108 Nice, France; xavier.noblin@unice.fr

**Keywords:** aggregation, oomycete, *Phytophthora* zoospores, potassium, carbonic anhydrase

## Abstract

Most pathogenic oomycetes of the genus *Phytophthora* spread in water films as flagellated zoospores. Zoospores perceive and produce signals attracting other zoospores, resulting in autoaggregation in vitro or biofilm formation on plant surface. The mechanisms underlying intercellular communication and consequent attraction, adhesion and aggregation are largely unknown. In *Phytophthora* *parasitica,* the perception of a K^+^ gradient induces coordinated motion and aggregation. To define cellular and molecular events associated with oomycete aggregation, we combined transcriptomic and ultrastructural analyses. Results indicate involvement of electroception in K^+^ sensing. They establish that the transcriptome repertoire required for swimming and aggregation is already fully functional at zoospore release. At the time points analyzed, aggregates are mainly constituted of zoospores. They produce vesicular and fibrillary material discharged at cell-to-cell contacts. Consistently, the signature of transcriptome dynamics during transition to aggregates is an upregulation of genes potentially related to vesicular trafficking. Moreover, transcriptomic and functional analyses show a strong enhancement of carbonic anhydrase activity, indicating that pH homeostasis may contribute to aggregation by acting on both zoospore movement and adhesion. This study poses the molecular and cellular bases of aggregative behavior within oomycetes and expands the current knowledge of ion perception-mediated dissemination of propagules in the rhizosphere.

## 1. Introduction

Oomycetes constitute a diverse group of eukaryotic microorganisms growing as filamentous coenocytic hyphae, reproducing both sexually and asexually and belonging, together with diatoms and brown algae, to the Stramenopiles [1]. Among oomycetes, several species of the genus *Phytophthora* cause highly destructive diseases on many dicots, with major ecological and economic worldwide consequences [2,3]. *Phytophthora* species spread in the environment as airborne sporangia or waterborne zoospores.

In water films, unicellular, biflagellate zoospores explore their environment and use sensory systems for the detection of stimuli such as chemical gradients (chemotaxis) and ionic fields (electrotaxis) [4]. They move towards the host plant and adhere to its surface to initiate infection. Colonization of the host surface may involve unicellular behavior or cell population dynamics through the formation of biofilms [5,6]. Once a small number of zoospores has reached the host surface, they produce signals that attract hundreds of individuals, resulting in biofilm formation of encysted spores [5,7]. The formation of such a structure may protect the pathogen from plant defense compounds or fungicidal treatments and enhance nutrient availability [5]. Following very short periods of flooding, the *P. parasitica* species is able to rapidly release zoospores, which remain motile for up to 20 h [8]. Repeated irrigations can disperse *P. parasitica* up to 70 m from the inoculum source [9]. Nevertheless, little is known about how zoospores communicate, coordinate their behavior and constitute an inoculum on the host, as studies have investigated this process at the multicellular level only recently [10,11].

Cell aggregation typically represents a transient life cycle stage of numerous eukaryotic clades [12]. Aggregative behavior has been described in different microbial eukaryotic lineages [13]. In the cases of the unicellular *Capsaspora owczarzaki* [12] and *Monosiga brevicollis* [14,15], genomic and transcriptomic studies revealed the presence of adhesion- and signaling-related proteins required for metazoan multicellular development, highlighting their putative involvement in the aggregative process in microbial eukaryotes [14,15]. In oomycetes, molecular mechanisms involved in signal perception and transduction leading to aggregation remain poorly understood. *Phytophthora* adhesive capabilities have been mainly investigated in terms of adhesion to the host ([16], for review). A recent study conducted in our laboratories showed that in vitro sensing of K^+^ by zoospores induces coordinated behavior and aggregation, possibly mediated by signaling events. Specifically, application of a K^+^ gradient to *P. parasitica* zoospores induces coordinated movement and rapid aggregation, as the result of a succession of negative chemotaxis and bioconvection events [7]. This finding may also have important implications in natural environment. In the rhizosphere, K^+^ gradients generated by soil–root exchanges would affect zoospores distribution and behavior. The ability of oomycete species to release millions of zoospores per single host plant [17], in combination with repulsion forces generated by soil particles and attraction generated by roots [7], would be determinant for the formation of aggregates/biofilms at the root surface.

Within the present study, transcriptomic, functional and ultrastructural microscopy analyses were integrated to propose a first definition of molecular patterns involved in signal perception, leading to oomycete collective movement and aggregation in response to K^+^.

## 2. Materials and Methods

### 2.1. Zoospore Suspension Preparation and K^+^ Gradient Generation

Mycelia of *P. parasitica* (isolate 310, *Phytophthora* INRA collection, Sophia Antipolis) were cultured and zoospores were produced as described by Galiana et al. (2019) [7], except for droplet volume that was increased to 1 mL. Ten microliters of 1-M KCl solution was applied in a local and oriented manner to the freshly prepared zoospores suspension, to reach a complete diffusion [7]. The experiment was performed in triplicates (R1, R2 and R3).

### 2.2. RNA Extraction and Sequencing

Samples were taken from control (not K^+^-treated cells; C) and K^+^-treated cells, at different time points (5, 10, 15 min; K5, K10, K15) after K^+^ application. At each time point, zoospores suspension (5*10^5^ cells/mL) taken from 12 droplets was transferred on ice, centrifuged at 5000 *g* for 5 min at 4 °C and collected as a pellet that was immediately transferred in liquid N_2_. The RNA was extracted using miRNeasy^®^ Mini Kit (Qiagen, Hilden, Germany). Quality and quantity of the extracted RNA were determined with NanoDrop (Thermo Fisher Scientific, Waltham, MA, USA) and Agilent Bioanalyzer, using RNA 6000 Nano Kit (Agilent Technologies, Santa Clara, CA, USA). Extracted RNA was then sequenced at the UCAGenomiX platform, Sophia Antipolis. First poly-A-containing mRNA were purified, the isolated molecules were fragmented, and a random-primed cDNA library was synthesized. Ligation of adaptors was performed followed by adaptor-specific PCR amplification. Paired-end sequencing was carried out with a read length of 2 × 75 pb using the Genome Sequencer Illumina HiSeq Technology (San Diego, CA, USA). Gene sequences were submitted to the NCBI with accession number GSE142435 (https://www.ncbi.nlm.nih.gov/geo/query/acc.cgi?acc=GSE142435), as part of the BioProject PRJNA597021.

### 2.3. Transcriptomic Analysis

Using the software STAR Tool (v. STAR_2.4.0a; [18]), the sequences were mapped against the *Phytophthora parasitica* INRA-310 reference genome, available at National Center for Biotechnology Information (NCBI; BioProject PRJNA259235). Each transcript was identified and quantified with featureCounts (v. subread-1.5.0-p3-Linux-x86_64; [19]). Expression levels were analyzed by tracking the mapped reads and by determining the Fragment Per Kilo base of exon per Million reads (FPKM) for each transcript. The medians of FPKM values were used to measure the gene expression level and to compare the C, K5, K10 and K15 samples. Statistical analysis to determine significance of obtained results were performed as described below. Heat maps representing expression levels and fold changes (FC) of significantly upregulated genes among the samples were drawn using Multiexperiment viewer (MeV) [20]. Gene annotation and data mining were first performed using the Pfam protein families database [21] and integrated with Gene Ontology enrichment Analysis (GOA; [22,23]) using QuickGO online tool (https://www.ebi.ac.uk/QuickGO/). List of GO terms corresponded to the *Phytophthora parasitica* INRA-310 reference proteome (release of the 17.07.2019) downloaded from http://geneontology.org/docs/download-ontology/. Candidate genes involved in electroception were individuated through reciprocal BLASTP analysis [24] against *L. erinacea* electrosensory cell channel sequences [25]. Genes of interest were then manually clustered accordingly. The highest expressed genes accounting for the 50% of total gene expression were manually assigned to COG/KOG categories [26]. Annotation of upregulated genes was integrated with InterProScan analysis [27], reciprocal BLASTP analysis against *Phytophthora* proteomes [24] and manual annotation and proteases were further annotated at the MEROPS database [28]. Distribution of upregulated genes sequences among different lineages was verified through BLAST analysis [24], setting E-value threshold to 1 × 10^−6^. Gene product topology and localization were predicted using the Protter tool [29], the TMHMM Server v. 2.0 (http://www.cbs.dtu.dk/services/TMHMM/) and the PSORT Prediction program [30].

### 2.4. cDNA Preparation and RT-qPCR Analysis

After a DNAse I treatment (Ambion, Austin, TX, USA), RNA (2 µg) was reverse-transcribed using SuperScript IV Reverse Transcriptase kit (Invitrogen, Carlsbad, CA, USA) and Applied Biosystems Veriti thermal cycler (Thermo Fisher Scientific, Waltham, MA, USA). RT-qPCR analyses were performed in technical duplicates, using 5 µL of cDNA diluted 1:20, SYBRGreen kit (Eurogentec SA, Seraing, Belgium) and AriaMx real-time PCR system (Agilent Technologies, Santa Clara, CA, USA). Gene-specific primer pairs were designed using primer3plus software [31]. Their specificity and efficiency were validated by the analysis of amplification profiles and dissociation curves. Based on previous studies conducted at different *P. parasitica* physiological stages [32,33,34], UBC and WS41 were selected as internal control genes and their stability was verify using geNorm (https://genorm.cmgg.be/). Primers sequences are available in Table S1 of Supplementary Materials (SM) .

### 2.5. Statistical Analysis

Regarding RNA sequencing data, statistical analysis was performed using R software [35], Bioconductor [36] packages, including either DESeq2 [37,38] or edgeR [39], and the SARTools package developed at PF2-Institut Pasteur [40]. Normalization and differential analysis were carried out according to both DESeq2 and edgeR models and packages. Nevertheless, for consistency—and considering the output of the analysis—we chose to discuss gene expression level and samples correlation results only according one of the two approaches, specifically edgeR. A Benjamini–Hochberg (BH) *p*-value adjustment was performed to take into account multiple testing and control the false positive rate to a chosen level (0.05) [41,42]. After normalization, genes for which the corresponding number of reads, was defined as “NA” (not available), where considered as not expressed.

Regarding RT-qPCR data, statistical analyses to identify genes harboring significant transcript abundance differences among the experimental conditions, was carried out using the SATQPCR TOOL software [43]. Statistical differences between all pairs of samples across all biological replicates were determined using pairwise *t*-tests with a pooled standard deviation, setting the *p*-value to 0.05 (** *p* < 0.01; * *p* < 0.05).

### 2.6. Scanning Electron Microscopy (SEM) and Transmission Electron Microscopy (TEM)

SEM and TEM were performed on zoospores previously fixed with 2.5% glutaraldehyde solution (1.6% for cell sections preparation) in 0.1-M sodium cacodylate buffer (pH 7.4, at room temperature) for 1 h (2 h for sections) and then stored at 4 °C.

For SEM, after 3 rinsing in distilled water, fixed zoospores were filtered on a 0.2 µm isopore filter. Samples on filters were subsequently dehydrated in a series of ethanol baths (70%, 96%, 100% 3 times, 15 min each). After a final bath in hexamethyldisilazane (HMDS, 5 min), samples were left to dry overnight. Samples on filters were mounted on SEM stubs with silver paint and coated with platinum (3 nm) prior to observations. SEM observations were performed with a Jeol JSM-6700F SEM (Akishima, Tokyo, Japan) at an accelerating voltage of 3 kV.

TEM analyses were performed on both whole cells and 80-nm-thin sections. Whole cells were prepared using the negative staining method. After 3 rinsing in distilled water, a drop of cells suspension (~10 µL) was left for 5 min on a TEM copper grid (400 mesh) with a carbon support film. The excess of liquid was removed with a filter paper. Subsequently, staining was done by adding a drop of 0.5% (*w*/*v*) aqueous solution of uranyl acetate on the grid for 1.5 min, followed by removal of excess solution.

For thin sections analysis, samples were rinsed in 0.1 M sodium cacodylate buffer and post-fixed in osmium tetroxide (1% in the same buffer) reduced with potassium ferrycyanide (1%), for 1 h. After a water wash, cells were dehydrated with several incubations at increasing concentrations of acetone, and embedded in epoxy resin (EPON). 80-nm-sections were contrasted with uranyl acetate (4% in water) and then lead citrate.

TEM observations were carried out with a JEOL JEM-1400 (Akishima, Tokyo, Japan) transmission electron microscope, operating at 100 kV and equipped with an Olympus SIS MORADA camera.

### 2.7. Protonography of Carbonic Anhydrase (*CA*) Enzymatic Activity

The detection of CA activity in gel was performed using protonography method described by [44] with the modifications detailed in SM.

### 2.8. Immunolocalization

Zoospores were fixed by mixing cell suspension with 3% paraformaldehyde (1:1) and incubation for 60 min on ice. After centrifugation (5000× *g* for 4 min at 4 °C) and washing with PBS, zoospores were spread onto glass slides and air-dry at 40 °C for 5 min. For intracellular detection, cells were permeabilized by adding PBS-Triton (0.1%) for 15 min at room temperature. Between 3 washing with PBS, samples were successively incubated for 1 h at room temperature: (i) with a blocking solution containing 5% of nonfat dry milk in PBS, pH 7.2, for 30 min; (ii) then with primary rabbit antibodies, diluted 1:100, and (iii) finally with a Fluoprobes 594-conjugated antibody (Interchim, Inc., Montluçon, France). A list of primary antibodies utilized is available in SM. Samples were then mounted in Fluoroshield (Sigma-Aldrich, St. Louis, MO, USA). Image acquisition were performed on the Microscopy Platform-ISA-INRAE 1355-UNS-CNRS 7254-INRAE PACA-Sophia Antipolis.

### 2.9. Verapamil Pharmacological Assay

Verapamil (50 µM) was applied to 100 µL droplets of freshly prepared zoospore suspension for 30 min, in order to test aggregation pharmacological inhibition. One microliter of 1-M KCl solution was applied to each droplet in a local and oriented-manner. Droplets prepared with KCl solution in absence of verapamil were used as negative control. Zoospore motion was captured after 5 and 10 min from K^+^ application (K5 and K10).

### 2.10. Image Analysis

Zoospore motion was investigated as previously described by Galiana et al. (2019) [7] using the FIJI TrackMate plugin. Videos were analyzed with the following parameters: estimated blob diameter, 12 µm and threshold, 10 µm; automatic initial thresholding; linking max distance, 20 µm; gap-closing max distance, 20 µm; gap-closing max frame, 2; spot filtering, duration of track above 2 s. For each remaining trajectory, we calculated the mean velocity. Based on the analysis of 50 individual immobilized cells, a speed of 7 µm/s was defined as the threshold below which cells were characterized as motionless.

## 3. Results

### 3.1. Ultrastructures of Zoospore Aggregates

**Figure 1:**
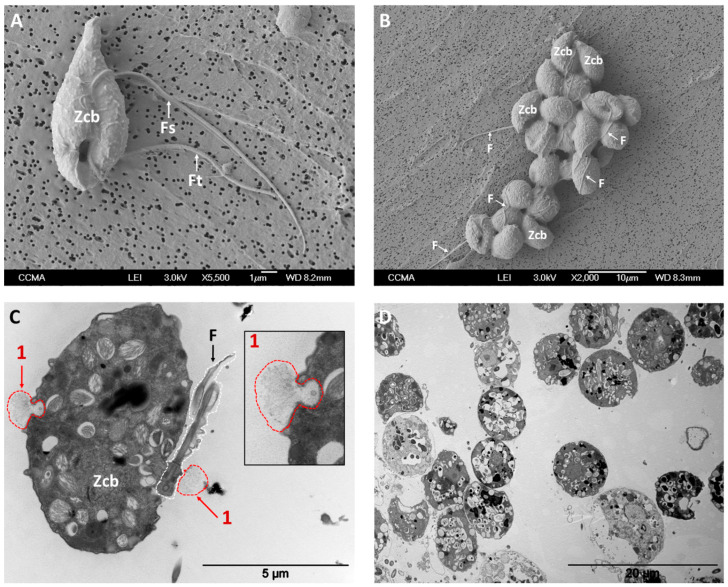
Micrographs of *P. parasitica* zoospores at unicellular stage, taken in absence of K^+^ (panels **A** and **C**) and of cell aggregates, taken at *t* = 15 min after K^+^ application (panels **B** and **D**). Zoospore cell bodies and flagella are indicated with “Zcb” and “F”, respectively. “Fs” and “Ft” indicate smooth and tinsel flagella, respectively. Panel C shows two secretory events (1); the inlet depicts a secretory vesicle that has just secreted its fibrillary content. Panel B shows the presence of several and representative ellipsoidal cells (such as those indicated as “Zcb”) and interwoven flagella (F), indicating a prominent proportion of cells at the zoospore stage in aggregates. Images were obtained through scanning electron microscopy (SEM; panels **A** and **B**) and transmission electron microscopy (TEM) on analyses of 80-nm-thin sections (panels **C** and **D**) in presence or absence of K^+^.

To further define cellular and molecular mechanisms implied in aggregation, K^+^ gradients were applied in vitro to freely swimming *P. parasitica* zoospores. The effect of the ionic flows on the zoospore behavior was monitored along time, showing a marked coordinated motion, with plume formation and downward migration (~10 min), resulting in aggregation (~15 min) (Figure 1 and Video S1).

Notably, K^+^-induced aggregates were composed of both encysted cells and zoospores, with flagella and cell bodies being interweaved (Figure 1B,D). Fifteen minutes after K^+^ treatment, the most of cells exhibited the characteristic ellipsoidal shape of zoospores rather than the circular conformation typical of cysts (Figure S1A and Video S2). Moreover, as previously observed by Galiana et al. (2019) [7], most of cells showed anticlockwise rotation movement or a short and erratic displacement indicative of the presence of flagella at that time point (Figure S1B and Video S2). Measurement of average speed, deduced from zoospore trajectories, indicated that 84% of cells moved at a speed higher than 7 µm/s, a threshold below which a cell was considered to be motionless and potentially encysted. This value decreased to 78%, when we considered a speed threshold of 10 µm/s (Figure S1C,D).

**Figure 2:**
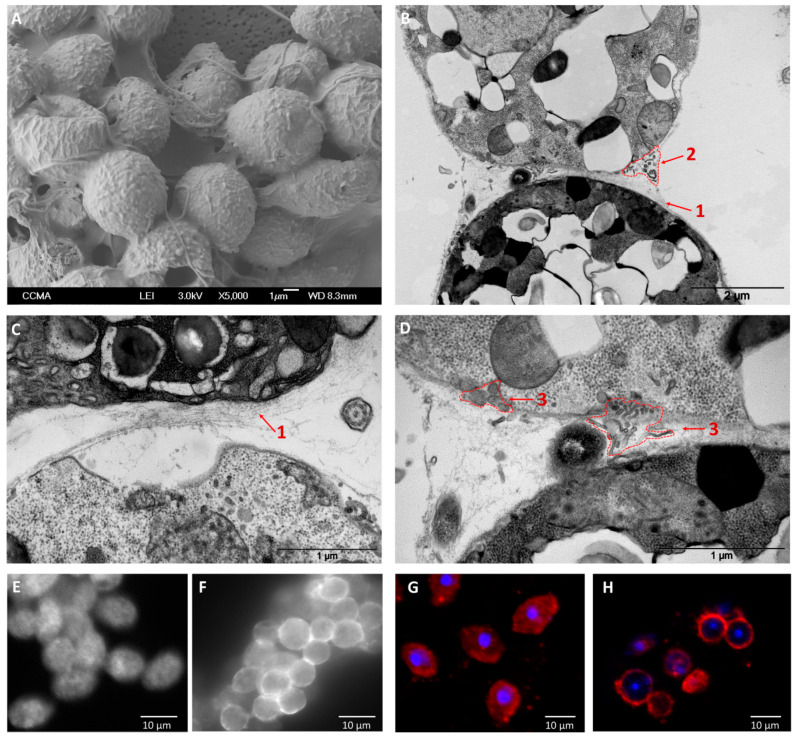
Ultrastructural and immunohistochemical observations of cell-to-cell contacts in K^+^-induced aggregates. Panels **A** and **B** provide a view of the developing extracellular matrix-like structure responsible for aggregation, observed on whole cells by scanning electron microscopy (SEM) and on 80-nm-thin sections by transmission electron microscopy (TEM) analyses, respectively. Panels **C** and **D** show details of the intercellular space observed on thin sections and unveiling (1) the accumulation of constitutive fibrillary material; (2) clusters of drop-shape and tubular structures, delineated by electron-dense material, adjacent to the outer side of the cell membrane (ranging, on average, from 69 ± 9 nm (*n* = 5) of vesicular structures to 229 ± 105 nm (*n* = 4) of tubular); or (3) being released from cell membrane (on average, from 41 ± 13 (*n* = 10) to 116 ± 25 nm (*n* = 4) for vesicular formations and 180 ± 52 nm (*n* = 2) for tubular). Panels **E**–**H** show immunohistochemical staining conducted with antibodies against human fibronectin (panels **E** and **F**) and protocadherin (FAT4) proteins (panels **G** and **H**), on *P. parasitica* zoospores that were not treated with K^+^ (panels **E** and **G**) or on K^+^-induced aggregates (panels **F** and **H**). In panels **G** and **H**, nuclei are stained with DAPI dye.

Conversely—and in agreement with a previous study [7]—application of other cations, such as Na^+^, in the same range of concentration, did not lead to zoospore aggregation (Video S3). Ultrastructural analyses conducted on K^+^-induced aggregates revealed the occurrence of cell-to-cell associations together with the deposition of intercellular matrix-like material (Figure 2A–D). This structure was characterized by the accumulation of fibrillary material connecting adjacent cells and delimiting the intercellular space (Figure 2B,C). Moreover, several clusters of vesicular and tubular structures were found adjacent to the outer side of the cell membrane or being released from cell membrane (Figure 2B,D). This is compatible with the liberation of vesicles from zoospores and the consequent secretion of material in the intercellular space, potentially involved in extracellular matrix-like release or, in communication and/or the onset of infection as reported by Zhang et al. (2013) [45]. Such liberation could also be visualized without K^+^ application from the outset of the zoospore stage, as illustrated in Figure 1C, showing exocytosis-mediated dorsal and ventral discharge of fibrillary materials from secretory vesicles into the water.

In a first attempt to analyze the composition of the material constituting the intercellular space, a targeted search on known adhesion and extracellular matrix molecules was conducted. An immunohistochemical approach was developed using antibodies raised against proteins involved in aggregation in lower eukaryotes, such as proteins related to fibronectins and protocadherins [12,46,47]. An anti-fibronectin antibody recognized antigens located at the intercellular space, and/or at cell membrane level, of K^+^-induced aggregates, decorating cell-to-cell contact areas (Figure 2F). In contrast, untreated zoospores only displayed faint and mostly diffused staining (Figure 2E).

The fibronectin staining clearly evoked the formation of adhesive structures, supporting the hypothesis of the recognition of the fibrillary material observed in Figure 2C. Moreover, the assay with antibodies raised against human FAT4 protocadherin showed a punctuated or diffused decoration of the cell body in untreated zoospores (Figure 2G), while K^+^-induced aggregates rather displayed a decoration of the cell perimeter (Figure 2H), although not specifically located at the intercellular space. According to these results, fibronectin-like proteins may be good candidates in the establishment of zoospore cell-to-cell aggregation, through extracellular matrix-like formation, while cadherin proteins should be involved in other adhesive functions.

### 3.2. Overview of the Transcriptome of Swimming Zoospores

RNA sequencing reads were assembled and mapped against the *P. parasitica* current genome assembly, which contains a total of 23,122 putative genes. Following normalization, 12,046 genes (52%) were expressed in swimming zoospores (C sample), accounting for 811,200 FPKM in total. Moreover, we noted that 10% of the total gene expression (TOP10) concerned only 11 genes, whose expression level ranged between 5549 and 12,281 FPKM. Similarly, 20 (TOP20) and 50% (TOP50) of the total data set resulted from the expression of 29 (3728 to 12,281 FPKM) and 137 (1193 to 12,281 FPKM) genes, respectively (data set S1).

Genes encoding ribosomal proteins and other proteins involved in translation were among the most expressed. On average almost 60% of the highly expressed genes belonged to the “translation” category, according to the KOG classification, with 6/11, 19/29 and 76/137 genes assigned to this function, among the TOP10, TOP20 and TOP50, respectively (Figure S2). A reciprocal BLAST analysis pointed out a homology of PPTG_13558, whose transcript was clustered among the 50% most expressed genes, with the *P. infestans* putative elongation factor 3 PITG_03712 [48]. Conversely, transcription activity appeared poorly represented, with only 2 genes annotated among the TOP50, indicating that mRNA repertoire required to zoospores would be already available before zoospore swimming.

Among the TOP50, the most expressed gene (PPTG_07912; 12,281 FPKM) lacked any homology to known sequences or known domain and was classified as “unknown”, as well as 15 other genes which remained without any functional annotation—or which turned out to correspond to transposable elements.

Notably, the second most expressed gene encoded a putative secreted elicitin-like protein (PPTG_13204; 11,304 FPKM). Extending the analysis to the first 137 most expressed genes revealed 7 genes possibly involved in virulence or organism defenses. Among them, an additional elicitin-like (PPTG_08950; [49,50]), a CRN effector (PPTG_07145; [51]), a TOS1-like glycosyl hydrolase effector (PPTG_16550; [52]), a cell-wall-degrading enzyme (PPTG_19098; [53]), a thioredoxin and a glutathione transferase (PPTG_08056 and PPTG_16940). Transcription of pathogenicity-related genes in zoospores, at such a high level, may indicate a dual role of these genes: an anticipation of *Phytophthora* requirements at early stages of the interaction with the host [48] or an alternate putative function related to the motile stage.

Moreover, the fifth and sixth most expressed genes presented respectively 2 alcohol dehydrogenase domains, containing a Zn^2+^ binding site (PPTG_17182; 6359 FPKM) and a ribonucleotide reductase domain (PPTG_16020; 6171 FPKM), possibly being involved in carbohydrate and nucleotide metabolism. Because of its role in DNA synthesis, ribonucleotide reductase could be involved in nuclear division after cyst germination [48]. Extending the analysis to the 137 most expressed genes identified 12 genes putatively involved in transport and metabolism of organic molecules. Among them, PPTG_08889 (2578 FPKM) encoded D1-pyrroline-5-carboxylate reductase (P5CR), the enzyme catalyzing the last step of proline biosynthesis [54]. P5CR is of particular importance for osmoregulation during zoospore release, as proline is an osmolyte expelled by the cell in hypoosmotic conditions [4].

Moreover, the seventh most expressed gene (PPTG_01661; 5845 FPKM) encoded the PnCcp protein, characterized by a Sushi domain, otherwise described as a complement control protein (CCP) module [55]. A previous study reported the secretion of PnCcp from large peripheral vesicles during *P. parasitica* zoospore encystment, with a possible involvement in adhesion to host surface, thanks to the presence of the Sushi adhesive domain [45].

Among the TOP20, 1 gene (PPTG_12173; 5538 FPKM) was annotated as a putative Zn^2+^ finger of class A20, otherwise characterized as an inhibitor of cell death and a stress-associated protein in plants [56,57]. Overall, very few transporters were identified among the TOP50, as only 5 genes potentially encoded two members of the PLAC8 family (PPTG_08418 and PPTG_03073), known to be involved in Cd metabolism in fungi and Ca^2+^ transport in plants [58,59], two mitochondrial carrier proteins (PPTG_07200 and PPTG_07274) and a member of the ATP-binding cassette (ABC) Superfamily of active transporters (PPTG_18357). Previous studies conducted on *P. infestans* reported the high expression of ABC encoding genes in zoospores, hypothesizing their involvement in pathogenicity [48]. In agreement with previous transcriptomic studies [48], 1 putative transcription factor, clustered among the TOP20 (PPTG_03306; 4287 FPKM), and 13 genes belonging to the TOP50 were assigned to signal transduction pathways, including protein kinases, phosphatases and phosphodiesterases.

### 3.3. Molecular Patterns Underlying Coordinated Behavior and Cell-to-Cell Adhesion of P. parasitica Zoospores

Microscopic analyses indicated that zoospore aggregation upon K^+^ application is a highly dynamic mechanism occurring in a very short time lapse. Thus, we anticipated that transcripts encoding adhesion proteins of the extracellular matrix would be already present in zoospores swimming freely. In particular, we searched for fibronectin-like and cadherin-like proteins in the data set. Seventeen expressed fibronectin-like genes, accounting for 174 FPKM in total, were identified, of which a single gene (PPTG_04004) accounted for 54 FPKM. Four of these 17 genes were predicted to have an extracellular or transmembrane location and exhibited repetitions characteristic of the fibronectin_3 (FN3) motif (PPTG_13378, PPTG_14266, PPTG_04587 and PPTG_01093). Additionally, we identified a low expressed gene (PPTG_11709) belonging to the oomycete-specific subfamily of tri-modular Nonagonal cadherins recently identified [60]. A total of 75 genes coding for adhesion molecules or putatively involved in extracellular matrix constitution were found, including the seventh most expressed gene (PPTG_01661, PnCcp) (data set S2). We then intended to identify genes otherwise shown to participate to external stimuli perception and subsequent coordinated behavior. To this aim, we mined transcriptomic data for genes relevant to osmoregulation, ion perception, homeostasis, as well as key signaling genes such as protein kinases or G protein-coupled receptors (GPCRs). We thus annotated 479 genes potentially involved in these functions (data set S3 and S4). It was interesting to note that these genes, which constitute 5% of the protein-coding gene content, take into account for 3% of the total FPKM amount in swimming zoospores, so that their representation in the data set may correspond to a basal expression level.

In order to identify genes relevant to zoospore electroception, whose molecular mechanisms remain unexplored, we looked for orthologs of genes described in animal and bacterial electrotactic systems, such as low-resistance channels (e.g., Big Conductance Ca^2+^-activated K^+^ (BK) channels and voltage-gated Ca^2+^ channels) [25,61]. We conducted reciprocal BLASTP analysis against channels from *Leucoraja erinacea* electrosensory cells [25] and retrieved in the *P. parasitica* transcriptome 13 distinct sequences putatively correlated to electroception (data set S3). Notably, the expression level of the most expressed BK channel encoding gene (PPTG_06455; 63 FPKM) did not differ from that of the one regulating the electrosensory system in *L. erinacea* cells (<100 FPKM; [25]). Conversely, L-type voltage-gated Ca^2+^ channels, characterized by the GPHH sequence, displayed poor expression, the highest expressed being PPTG_06417 (2 FPKM). Despite the low expression of the corresponding genes, a pharmacological assay conducted with verapamil, which is known to block voltage-dependent Ca^2+^ channels, resulted in the inhibition of zoospore aggregation, in presence of K^+^ (K10) (Figure S3A,B). Moreover, measurement of verapamil-treated zoospore mean speed, 5 min after K^+^ application (K5) indicated that the inhibition of aggregation did not result from an impairment of the zoospore motion (Figure S3C). In addition, the inhibitory activity of verapamil on voltage-gated K^+^ channels led us to hypothesize a role of these channels in K^+^-mediated aggregation. We subsequently identified 10 expressed genes encoding voltage-gated K^+^ Channel β subunits in the *P. parasitica* transcriptome (data set S4). These results support the hypothesis of a role of voltage-gated K^+^ and Ca^2+^ channels and therefore BK channels, in the K^+^ upstream perception event and/or establishment of K^+^-induced aggregation.

### 3.4. P. parasitica Zoospore Transcriptome Dynamics and Cell Response to K^+^ Gradient Application

The overall transcriptomic analysis showed that most of genes were equally represented among the different experimental conditions. The amount of genes annotated in samples, taken at different times after K^+^ gradient application—and the putative functions that they encoded—did not remarkably differ from those of freely swimming zoospores, with 10 genes constituting the TOP10 of total gene expression, and 27 ± 1 and 130 ± 1 genes representing the TOP20 and TOP50, respectively (Figure S4).

**Figure 3:**
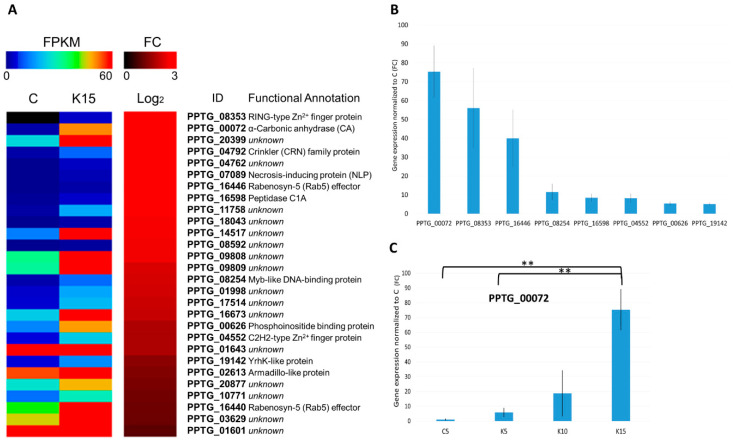
Overview of upregulated genes. (**A**) Heat map of expression levels (Fragment Per Kilo base of exon per Million reads, FPKM; left part of the panel) and fold change (FC, log2; right part of the panel) of significantly upregulated genes (defined by their ID and putative functional annotation) upon K^+^ application (K15 sample) compared to freely swimming zoospores (C sample). Only genes with FC > 2 (log2 > 0) were considered. Correspondence between colors and FPKM or FC is reported in the scale at the top of each panel; (**B**) expression of a selection of upregulated genes, analyzed by RT-qPCR and expressed as FC in K15 sample compared to C. Panels **C** shows the differential expression of Carbonic Anhydrase (CA) PPTG_00072, expressed as FC, at different times after K^+^ application (K5, K10, K15), compared to the C sample, analyzed by RT-qPCR. ** *p* < 0.01.

Despite important ultrastructural changes, such as deposition of material in the intercellular space, occurring in zoospores undergoing aggregation, statistically significant variation in gene expression among the 4 tested conditions concerned only a very restricted number of genes. The number of genes whose expression was modulated upon K^+^ gradient application was extracted using the edgeR package and is provided in Table S2. According to the statistical method applied to RNA sequencing data and after validation by RT-qPCR, 28 genes were found to be significantly upregulated (up to 33-fold) in K15 compared to the C sample (Figure 3, Figure S5 and data set S5). A change in the transcriptional program was obvious in K15 sample, not only compared to the control, but also to the samples collected at earlier stages after K^+^ application (K5 and K10), with 11 and 21 upregulated genes, respectively, the most being the same found when comparing C vs. K15. A complete overview of the upregulated genes among the different conditions is provided in data sets S5–S7. This result is consistent with a progressive diffusion of the K^+^ gradient, ensuring a higher contact between K^+^ and zoospores after 15 min compared to earlier time points. Therefore, the change in the transcription program, induced by K^+^, can be seen as a gradual and incisive process, involving a well-defined set of genes. No gene was found to be significantly downregulated upon K^+^ application.

#### 3.4.1. Overview of K^+^-Induced Transcriptome Dynamics

List and expression levels of the 28 genes upregulated in K15 compared to the C sample is provided in Figure 3, Figure S5 and data set S5. From the analysis of each gene expression level, PPTG_08353 was the most upregulated gene, increasing >33-fold upon K^+^ treatment. This gene possesses a RING-type Zn^2+^ finger domain and a PX domain. Notably, the combination of these two domains was found to be specific of Oomycetes. While the RING finger domain is possibly involved in protein–protein interactions [62], the PX domain binds phosphatidylinositol molecules [63], which mediates the anchoring of protein to cell membranes. Additionally, the PX domain is known to play a role in protein trafficking and vesicular fusion, and is found in SNARE-related proteins, which regulate selective membrane fusion during vesicular release [63]. Therefore, PPTG_08353 could be potentially involved in signaling and/or the release/regulation of secreted material such as adhesion proteins. Similarly, PPTG_00626 (increased >3-fold; 50 FPKM) presented also a PX domain, possibly regulating the same processes. Among the other upregulated genes containing Zn^2+^ finger domains, 2 oomycetes specific genes, PPTG_16440 and PPTG_16446, display analogies with Rabenosyn-5 (Rab5). Rab5 effector is recruited to early endosomes in a phosphatidylinositol 3-phosphate kinase (PI3PK) dependent manner, insuring efficient trafficking [64]. The second most upregulated gene encodes a putative extracellular carbonic anhydrase (CA), specifically an α-CA enzyme (PPTG_00072), upregulated >29-fold in K15 sample. Conversely, the third highest upregulated gene (PPTG_20399; >18-fold), a *Peronosporales* specific sequence, encoding a putative intracellular protein and accounting for 345 FPKM in K15, escaped to a formal functional annotation, as well as several other most expressed genes (PPTG_03629, PPTG_09809, PPTG_09809, PPTG_02613, PPTG_01601 and PPTG_01643; 107 to 379 FPKM), despite the prediction of cytoplasmic or extracellular locations. Notably, 2 genes potentially related to pathogenicity, displayed a 10- and 9-fold change. These genes, namely PPTG_04792 and PPTG_07089 encode two effectors, a cytoplasmic CRN and an apoplastic Necrosis-inducing protein (NLP) [51,65]. We also identified a gene, PPTG_16598 (7-fold change) which was further annotated as MER1164209 in the MEROPS database, which corresponds to a cysteine protease (peptidase) belonging to the subfamily C1A [66]. Upregulation of putative transcription factors was also noted as a trait of zoospore response to K^+^. PPTG_04552, increasing >3-fold, presents 4 C2H2-type Zn^2+^ finger domains, the most common eukaryotic DNA-binding motif functioning as transcription factor, but also able to bind RNA and proteins [67]. Moreover, PPTG_08254, increasing >4-fold, presents a SANT domain that, despite its high similarity with Myb-like DNA-binding domain, would be rather involved in chromatin remodeling, functioning as histone-binding module [68]. Upregulation of these genes could play a role in enhancing the transcription of specific genes, promoting K^+^-induced zoospore aggregation.

#### 3.4.2. A Carbonic Anhydrase (CA) Is Strongly Induced upon K^+^ Treatment

**Figure 4:**
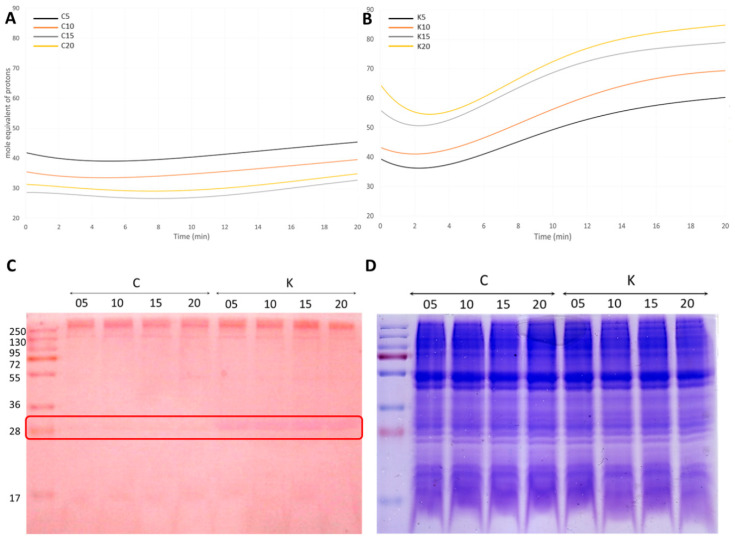
Zoospore carbonic anhydrase (CA) enzymatic activity assay. Whole cell extracts were prepared at each time point (5, 10, 15, 20 min) without (C for control) or with a K^+^ gradient application (K). Upper panels (**A**) and (**B**) show CA enzymatic kinetics at different time points posttreatment, for controls or K^+^-treated cells. The amount of product formed is plotted as a function of time along the CA assay. Lower panels (**C**,**D**) show protonography analysis of CA enzymatic activity performed using neutral red as indicator of proton amount and Blue Coomassie staining as positive control for revelation of total protein patterns, respectively.

One of the hallmarks of the transcriptomic response to K^+^ is the upregulation of PPTG_00072 (29-fold in K15 sample compared to C) encoding for a putative extracellular α-CA enzyme (Figure 3A,B and data set S5). Moreover, comparing K15 to K5 and K10 samples revealed that this gene displayed a 12 and 13-fold induction, respectively, pointing out a step-wise increased gene expression, notably before aggregation when zoospores swarmed and then formed a plume (Figure 3C and data set S6 and S7). As expected, the predicted protein contained the typical signature for Zn^2+^ binding site (GO: 0008270) and carbonate dehydratase activity (GO:0004089), which mediates the interconversion of CO_2_ and water to HCO_3_^-^ and H^+^ and, thus, regulate acid–base cell homeostasis [69]. Consistent with RNA-Seq and RT-qPCR analyses, a CA staining gel assay revealed the accumulation in K^+^ samples of a protein with an apparent molecular weight (mw) of ~28 kD, close to the expected mw of α-CA (28,405.61 Da, Figure 4C). According to the analysis of enzymatic kinetics shown in Figure 4A,B, CA activity remained quite stable and low in control samples, while it showed a prominent activity detected as soon as 5 min after K^+^ exposure, and increased progressively in the following time points analyzed. Such increase appeared strictly correlated with the increase of PPTG_00072 transcript accumulation upon K^+^ treatment. A CA staining gel assay was also performed in the presence of a Na^+^ gradient, at the same range of concentration as used with K^+^ and that did not induced aggregation, at different time points (5 to 20 min). This assay showed a lower and quite stable activity, comparable to that of control sample, indicative of a K^+^ specific effect on CA activity in correlation with the sequence of events preceding and occurring during aggregation (Figure S6A).

Finally, besides PPTG_00072, data mining revealed the presence in the *P. parasitica* transcriptome of 5 other expressed genes annotated as putative CAs, having either a cytosolic or an extracellular location (data set S8). Among them, only PPTG_00072 showed an upregulation, and was the most expressed (>50 FPKM in K15), while the other genes were only poorly represented.

## 4. Discussion

Cellular aggregation was described within several eukaryotic clades as a life stage of the organism, typically emerging under starvation conditions and representing a selective advantage, as it enhances the contact with the target host and the access to nutrients [12,13].

Consistent with previous studies conducted on bacteria and other *Phytophthora* species [7,61,70], the rapid zoospore aggregation observed in this study states the effect of K^+^ gradient on *P. parasitica* coordinated behavior and reinforces the involvement of electro- and/or chemo-reception in K^+^ flow sensing and zoospore aggregation. As suggested by the verapamil pharmacological assay, inhibition of voltage-gated Ca^2+^ channels, which are responsible for the activation of BK channels [25], resulted in inhibition of zoospore aggregation, but not motility impairment. This result suggests a role of electroception, mediated by voltage-gated Ca^2+^ channel activation, in K^+^ sensing and K^+^-induced aggregation. Moreover, this result points out a role of these channels, known to be involved in zoosporogenesis, encystment and germination [48,71,72], also at the swimming zoospore stage.

K^+^ application leads to zoospore plume formation, downward migration and aggregation on the support surface. For this reason, in the present study, cell adhesion was considered for the first time in the context of the establishment of cell-to-cell aggregation, rather than being limited to adhesion to the plant target. The analysis of cell-to-cell associations revealed in *P. parasitica* the accumulation of a fibrillary structure resembling an extracellular matrix, connecting adjacent cells and delimiting the intercellular space. Moreover, the release of vesicular structures led us to hypothesize a concerted cellular response to K^+^ gradient implying the secretion of extracellular matrix-like material and eventually factors involved in cell-to-cell communication and infection establishment. Based on a study on *C. owczarzaki*, showing involvement of fibronectin_3 (FN3) domain in cell aggregation [12], we hypothesized that *P. parasitica* proteins harboring a high number of FN3 repeats may mediate cell-to-cell adhesion. Additionally, an oomycete-specific cadherin family was recently identified [60] and a role of these molecules in signal transduction in choanoflagellates as well as adhesion and aggregation establishment in *Dictyostelium* and vertebrates was previously reported [46,47]. The immunohistochemical approach applied here revealed fibronectin decoration of aggregate intercellular material, supporting the hypothesis of the involvement of such proteins in extracellular matrix-like formation. Additionally, cadherin staining of the cell membranes connects its possible functions to cell adhesion.

The drastic, phenotypic ultrastructural changes resulting in aggregate formation was accompanied by a very limited, although sharp, modification of the transcriptome at the qualitative level. Within the fraction of expressed genome (12,046 genes, 52%), the 50% of total gene expression covered only 137 genes, which represent only 1% of expressed genes, pointing out a predominance of genes relevant to the translation machinery, including ribosomal proteins. Conversely, molecules typically involved in metazoan cell signaling, adhesion and extracellular matrix formation such as cadherin, fibronectin, laminin and integrin, tyrosine kinases and GPCRs, previously observed in choanoflagellates [14,15], constituted only 3% of total gene expression (554 clustered genes). These results brought us to assume that, in *P. parasitica*, aggregation would be mediated by still uncharacterized mechanisms. Moreover, upregulation of genes putatively involved in vesicular trafficking could explain the release of vesicular material observed within extracellular material surrounding zoospore aggregates.

Transcriptomic data taken together with enzyme kinetics and molecular analyses converge toward the hypothesis of a cellular response to K^+^ application involving extracellular CA activity. Hence, enzymatic assays revealed an induction of PPTG_00072 in an ion specific manner upon K^+^, and not Na^+^ gradient, correlating the CA activity to aggregation establishment.

CA enzymes are implicated in several important biological processes. A combination of cytoplasmic and membrane-associated CAs are involved in erythrocyte CO_2_ take up/release [73]. Interaction of CA with transporters, aiming at maintaining pH homeostasis, was observed in several mammalian cells and tissues, including kidney, pancreas and heart [74], retina [75] and several forms of cancer [76]. Based on the present study, we hypothesize a functional association among the extracellular PPTG_00072, uprising upon K^+^ gradient sensing, cytosolic CAs and an ion transporter, with CAs actively participating to pH homeostasis. In a prototypical cell, pH is regulated by a set of acid extruders and acid loaders [77]. Among them, few families were found within the *P. parasitica* transcriptome, specifically V-ATPases, Na^+^/H^+^ exchangers and Proton-dependent Oligopeptide Transporters (POT) (data sets S3 and S4). A possible candidate acting in association with CA(s) could be the V-ATPase complex, involved in zoospore osmoregulation through H^+^ transport into spongiome and H_2_O release from the water exclusion vacuole (WEV) [78]. Upon K^+^ sensing, the V-ATPase-CA association could regulate vesicular acidification, as observed in vertebrates during the pH-dependent bone resorption process involving intracellular CA II [79]. A second set of candidates could be the 12 expressed genes encoding putative Na^+^/H^+^ exchangers. Na^+^/H^+^ exchangers were previously found functionally associated with CA II, ensuring intracellular pH maintenance in heart muscle stretch [80].

## 5. Conclusions

Determination of transcriptomic and ultrastructural signatures revealed that K^+^-induced aggregation is mediated by an electrotactic response to K^+^ gradient. For aggregation, zoospores recruit preformed extracellular matrix-like proteins and respond by gene expression for vesicular trafficking and pH homeostasis. This work could help expanding the current knowledge of aggregative multicellular behavior of microorganisms, by posing the bases of a first model of propagule response to ion perception within oomycetes. By defining zoospore behavior in response to a major soil mineral component such as K^+^, this study also contributes to delineate the complex network of biotic and abiotic interactions occurring in the environment and driving the distribution of oomycete propagules.

## Supplementary Materials

The following are available online at https://www.mdpi.com/2076-2607/8/7/1012/s1, Supplementary File, data sets S1–S8 and Videos S1, S2 and S3. Video S1: Zoospores forming a plume upon K^+^ treatment (t = 12 min) and migrating downward forming an aggregate on the support surface. The downward migration toward the aggregate location is visualized through displacement of the microscope stage for 12 s. Video S2: Behavior and shape of cells observed 15 min after K^+^ treatment. The sequence shows an area of moderate cell density where most of cells exhibit the ellipsoidal shape typical of zoospores and an anticlockwise rotation movement indicative of the presence of flagella attached to the cell body at that time point. Video S3: Zoospore behavior upon Na^+^ gradient application (t = 12 min). No effect on zoospore swimming patterns and parameters, such as speed and trajectory, is observed, in presence of Na^+^ applied in the same range of concentrations than K^+^. Na^+^ was not found to induce zoospore aggregation in these conditions.
