# Supplementary material for: Transcriptomic and Ultrastructural Signatures of K+-Induced Aggregation in Phytophthora parasitica Zoospores
Source: Microorganisms. 2020 Jul 7;8(7):1012. doi: 10.3390/microorganisms8071012 (PMC7409359; doi:10.3390/microorganisms8071012)
Supplement: Supplementary file 1 [file microorganisms-08-01012-s001.zip › Supplementary File_microorganisms-834967_revised.doc]

**Supplementary File**

**Transcriptomic and ultrastructural signatures of K^+^-induced aggregation in *Phytophthora parasitica* zoospores**

Ilaria Bassani ^1,^*, Corinne Rancurel ^1^, Sophie Pagnotta ^2^, François Orange ^2^, Nicolas Pons ^3^, Kevin Lebrigand ^3^, Franck Panabières ^1^, Laurent Counillon ^4^, Xavier Noblin ^5^ and Eric Galiana ^1,^*

^1^	Université Côte d'Azur, INRAE, CNRS, ISA, 06903 Sophia Antipolis, France; [corinne.rancurel@inrae.fr](mailto:corinne.rancurel@inrae.fr) (C.R.); [franck.panabieres@inrae.fr](mailto:franck.panabieres@inrae.fr) (F.P.)

^2^	Université Côte d'Azur, Centre Commun de Microscopie Appliquée, 06108 Nice, France; [sophie.pagnotta@unice.fr](mailto:sophie.pagnotta@unice.fr) (S.P.); [francois.orange@unice.fr](mailto:francois.orange@unice.fr) (F.O.)

^3^	Université Côte d'Azur, CNRS, IPMC, 06560 Sophia Antipolis, France; [pons@ipmc.cnrs.fr](mailto:pons@ipmc.cnrs.fr) (N.P.); [lebrigand@ipmc.cnrs.fr](mailto:lebrigand@ipmc.cnrs.fr) (K.L.)

^4^	Université Côte d'Azur, CNRS, LP2M, 06107 Nice, France; [laurent.counillon@unice.fr](mailto:laurent.counillon@unice.fr) (L.C.)

^5^	Université Côte d'Azur, CNRS UMR 7010, Institut de Physique de Nice, 06108 Nice, France; [xavier.noblin@unice.fr](mailto:xavier.noblin@unice.fr) (X.N.)

*****	Correspondence: [ilaria.bassani@inrae.fr](mailto:ilaria.bassani@inrae.fr) (I.B.); [eric.galiana@inrae.fr](mailto:eric.galiana@inrae.fr) (E.G.)

**This Supplementary File includes:**

Supplementary Materials and Methods

Figures S1-S6

Tables S1-S2

**Supplementary Materials and Methods**

*Modifications to Protonography of Carbonic Anhydrase (CA) enzymatic activity*

Whole zoospore extracts were prepared in the Laemmli sample buffer (60 mM Tris-Hcl buffer (pH 6.8) containing 2% SDS and 0.004% bromphenol blue) without addition of reducing agent. Extracts (5 µg proteins) were subjected to a 12% polyacrylamide gel electrophoresis by running at 100 V. The gels were successively washed twice in 10 mM Tris-HCl buffer (pH 7) with 0.5% triton, 10% isopronol, washing buffer, and 0.5% neutral red for 30 min. To visualize CA enzymatic activity the gels were immersed in CO_2_-saturated H_2_O in the presence of 1mM ZnCl_2_, prepared by dropping 10 g of dry ice to 200 mL of deionized H_2_O (18 MΩ) and incubated at 20°C. CA activity kinetics were performed thought a time sequence analysis of the change of proton concentrations from the time point at which each gel was immersed in CO_2_-saturated H_2_O. Variations in neutral red dye at the band corresponding to the molecule exhibiting enzymatic activity were quantified. Regions of Interest (ROIs) were defined and binary images were generated using ImageJ time_series_analyzer plugin, to determine the number of particles at each time point (frame rate equal to 33.3 images/sec).

*List of primary antibodies utilized in Immunolocalization*

The following primary antibodies were used:

- a rabbit Protocadherin FAT4 polyclonal antibody (Elabscience, Houston, TX, USA);
- a rabbit Fibronectin monoclonal antibody (Epitomics, Nanterre, France).

**Table S1.** Sequence of target and housekeeping genes primers utilized in RT-qPCR analysis.

| **Gene** | **Primer** | **Sequence 5’ 🡪 3’** |
| --- | --- | --- |
| **UBC** | F | CCACTTAGAGCACGCTAGGA |
|  | R | TACCGACTGTCCTTCGTTCA |
| **WS41** | F | TTCAAGTCCAGTGAGATCGG |
|  | R | TTGTGTCTTTGTGTGATGCG |
| **PPTG_00072** | F1 | ACGAAATGGGAGTCCATGAG |
|  | R1 | GTTTTTGAACCACCCACCAG |
| **PPTG_00626** | F1 | CAGTTGGCGTGATCATCTTC |
|  | R1 | GAATAGCACTTGGCGAGGAG |
| **PPTG_04552** | F1 | AGCCTCATCAATGCCCTATC |
|  | R1 | TGTCCTTGCTCGTGAAGATG |
| **PPTG_08353** | F1 | TAACCCTTCCGTGTAAGCACTC |
|  | R1 | ACCATTGCTCCTGCTTCTTC |
| **PPTG_09808** | F1 | GTTCACTATTCCCCCTTCCAC |
|  | R1 | TTGTCATTCGGAGAGGAACC |
| **PPTG_16446** | F1 | CACGTTGTGTTTCGCTTGTC |
|  | R1 | CACCCTGGTGATCTTAATGTCC |
| **PPTG_16673** | F1 | AACGTGCGGTCAAACAAGAG |
|  | R1 | ATGACACTGCAATCGCTCAC |
| **PPTG_19142** | F1 | CCAGTTGGTGTTTTGTCGTG |
|  | R1 | TGGAGGATGCCATGTAGTTG |
| **PPTG_08254** | F1 | CTCTATCGCCGCCTTTATTG |
|  | R1 | GGGCGTACGAATTTCTTCTTC |
| **PPTG_16598** | F1 | CTTCATGAGCAAGCAGGATG |
|  | R1 | TGTGTTGCCTCGAACGTATG |
| **PPTG_13205** | F1 | TTCGGCGCTAACTGAAGAG |
|  | R1 | TCGAAAGGGTCCAGAATGTC |
| **PPTG_13206** | F1 | GATGGTACCGTCGTTTTTGG |
|  | R1 | TCCTGCATCATTTGAGCATC |

**Figure S1:** Panels A and B show respectively shape and behavior of cells 15 min after K^+^ treatment. Most of cells exhibit the characteristic ellipsoidal shape of zoospores rather than the circular conformation typical of encysted cells. Moreover, the majority of cells describe short but non-zero displacement, indicative of the presence of flagella at this time point. Panels C and D report mean speed percentile in K^+^-treated zoospore at t=15 min. For the 84% of cells, the speed deduced from zoospore trajectories is higher than 7 µm/s (green area of panel C), speed below which a cell is considered to be motionless and potentially encysted (red area of panel C). Percentage of motile cells decreases to 78%, if 10 µm/s speed threshold is considered.

**Figure S2:** Distribution of genes among defined COG/KOG categories within different ranges of expression level: (A) 10% of total gene expression (expressed as Fragment Per Kilo base of exon per Million reads (FPKM) and concerning 11 genes), (B) 20% of total gene expression (29 genes) (C) 50% of total gene expression (137 genes).

**Figure S3:** Aggregation pharmacological inhibition assay in presence of verapamil. (A) Droplets of freshly prepared zoospore suspension displaying aggregative behavior, 10 min after K^+^ application (K10). (B) Droplets treated with verapamil (50 µM), 30 min prior to K^+^ application, showing inhibition of K^+^-induced aggregation. The experiment was performed in replicates (n=6). (C) Zoospore mean speed, measured from replicate samples (n=3), after 5 min K^+^ application (K5), in presence or absence of verapamil (50 µM; 30 min post verapamil treatment). The graph indicates that the inhibition of aggregation displayed in panel B is not determined by an effect of Verapamil on zoospore motility.

**Figure S4:** Distribution of genes at different ranges of expression level, i.e. number of genes accounting for 10, 20 and 50% of total gene expression (expressed as Fragment Per Kilo base of exon per Million reads (FPKM)), across the different experimental conditions (absence or presence of K^+^ at different time points).

**Table S2.** Number of upregulated and downregulated genes for each condition comparison performed with edgeR package.

| **Condition comparison** | **Upregulated genes** | **Downregulated genes** |
| --- | --- | --- |
| **K5 vs C** | 0 | 0 |
| **K10 vs C** | 0 | 0 |
| **K10 vs K5** | 0 | 0 |
| **K15 vs K10** | 11 | 0 |
| **K15 vs K** | 21 | 0 |
| **K15 vs C** | 28 | 0 |

**Figure S5.** Expression of genes upregulated 15 min after K^+^ application (K15 sample), compared to freely swimming zoospores (C sample), analyzed by RT-qPCR and expressed as fold change (FC).

**Figure S6:** Protonography analysis of Carbonic anhydrase (CA) enzymatic activity performed using neutral red as indicator of proton amount (A), and Blue Coomassie staining as positive control for total cell extract proteins (B), for zoospores swimming freely (C sample) and submitted to K^+^ or Na^+^ gradient, at different time points (5, 10, 15, 20 min post-treatment).
